# Detection of scene-relative object movement and optic flow parsing across the adult lifespan

**DOI:** 10.1167/jov.20.9.12

**Published:** 2020-09-18

**Authors:** Lucy Evans, Rebecca A. Champion, Simon K. Rushton, Daniela Montaldi, Paul A. Warren

**Affiliations:** Division of Neuroscience & Experimental Psychology, School of Biological Sciences, Faculty of Biology, Medicine and Health, University of Manchester, Manchester, UK; School of Psychology, Cardiff University, Cardiff, UK

**Keywords:** flow-parsing, optic flow, motion perception, healthy ageing

## Abstract

Moving around safely relies critically on our ability to detect object movement. This is made difficult because retinal motion can arise from object movement or our own movement. Here we investigate ability to detect scene-relative object movement using a neural mechanism called *optic flow parsing*. This mechanism acts to subtract retinal motion caused by self-movement. Because older observers exhibit marked changes in visual motion processing, we consider performance across a broad age range (*N* = 30, range: 20–76 years). In Experiment 1 we measured thresholds for reliably discriminating the scene-relative movement direction of a probe presented among three-dimensional objects moving onscreen to simulate observer movement. Performance in this task did not correlate with age, suggesting that ability to detect scene-relative object movement from retinal information is preserved in ageing. In Experiment 2 we investigated changes in the underlying optic flow parsing mechanism that supports this ability, using a well-established task that measures the magnitude of globally subtracted optic flow. We found strong evidence for a positive correlation between age and global flow subtraction. These data suggest that the ability to identify object movement during self-movement from visual information is preserved in ageing, but that there are changes in the flow parsing mechanism that underpins this ability. We suggest that these changes reflect compensatory processing required to counteract other impairments in the ageing visual system.

## Introduction

The capacity to detect and assess movement in the environment during movement of the observer is critical to safe activity. Without this capacity, every time an observer moved, movement in other parts of the scene would be overlooked or incorrectly interpreted, leading to potentially inappropriate actions in response to that environmental movement. Consider, for example, the importance of rapidly detecting and accurately estimating the scene-relative movement of other cars when driving, or a dog that suddenly crosses your path while cycling. Here we investigate whether these abilities decline in healthy ageing, a question that is particularly relevant given previous well-established results demonstrating impaired motion processing in this group.

Both detection and assessment of movement in the environment are made difficult during self-movement because observer movement leads to complex patterns of visual motion (referred to here as *optic flow*). The addition of such patterns across the entire visual field means that retinal motion of objects is not directly related to their scene-relative movement. More specifically, observer movement and the resultant optic flow typically causes scene-stationary objects to move on the retina and scene-moving objects to have retinal trajectories that are inconsistent with their movement in the scene. To accurately judge scene-relative object-movement, the brain must parse the retinal input to identify and compensate for (i.e., filter out) the components of retinal motion due to self-movement (Rushton & Warren, 2005).

A considerable body of research over the last 70 years has examined compensation for the retinal consequences of observer movement using extra-retinal cues. This work has considered both eye rotation during pursuit eye movements and translation of the eye through space. Particular emphasis has been placed on the role of efferent motor commands in such accounts (von Holst & Mittelstaedt, 1950; Freeman & Banks, 1998; Freeman, 1999; Freeman, Banks, & Crowell, 2000; Wexler, 2003; Tcheang, Gilson, & Glennerster, 2005; Garzorz, Freeman, Ernst, & MacNeilage, 2018). More recently, further work has considered the additional role of vestibular information (Gogel, 1990; Li & Angelaki, 2005; Butler, Smith, Campos, & Bülthoff, 2010; MacNeilage, Zhang, DeAngelis, & Angelaki, 2012; Niehorster & Li, 2017) and/or proprioceptive feedback (Wallach, 1985; Wallach, Stanton, & Becker, 1974; Hlavacka, Mergner, & Bolha, 1996; Wexler, 2003; Tcheang et al., 2005).

A more recent set of studies has focused on the use of visual information to compensate for the retinal consequences of observer movement. The flow parsing hypothesis (Rushton & Warren, 2005; Warren & Rushton, 2009a) proposes that compensation for retinal motion arising during observer movement could also occur on the basis of our well-established sensitivity to optic flow. To see that this purely visual solution is necessary, consider the example of passive movement through the environment at constant speed (e.g., during motorway driving). In such circumstances nonvisual cues to self-movement are either absent or significantly degraded, and yet we are still able to identify movement of other objects in the environment. Under the flow parsing hypothesis, it is argued that the brain uses its sensitivity to optic flow to identify and parse out the components of retinal motion due to observer-movement—i.e., perform something akin to a global subtraction of the optic flow component of retinal motion. If complete subtraction of optic flow were possible then any remaining retinal motion would be due, unambiguously, to scene-relative movement of other objects in the environment. Evidence for the existence of an optic flow parsing mechanism that performs exactly this kind of global subtraction operation has now been presented by several groups, across many studies, using varied experimental techniques (Rushton & Warren, 2005; Warren & Rushton, 2007; Rushton, Bradshaw, & Warren, 2007; Warren & Rushton, 2008; Warren & Rushton, 2009a; Warren & Rushton,2009b; Matsumiya & Ando, 2009; MacNeilage, Zhang, DeAngelis, & Angelaki, 2012; Warren, Rushton, & Foulkes, 2012; Rushton, Foulkes, & Warren, 2013; Foulkes, Rushton, & Warren, 2013b; Fajen & Matthis, 2013; Layton & Fajen, 2016; Niehorster & Li, 2017; Rogers, Rushton, & Warren, 2017; Rushton, Niehorster, Warren, & Li, 2018; Rushton, Chen, & Li, 2018).

Given the potential functional significance of flow parsing it is important to consider groups that might be particularly prone to impairment in this mechanism, e.g. because of altered motion perception. Healthy older observers represent one such group, exhibiting a multitude of impairments in motion processing (Tran, Silverman, Zimmerman & Feldon, 1998; Betts, Taylor, Sekuler and Bennett, 2005; Tadin & Blake, 2005; Snowden & Kavanagh, 2006; Bennett, Sekuler & Sekular, 2007; Billino, Bremmer & Gegenfurtner, 2008; Kavcic, Vaughn & Duffy, 2011; Hutchinson, Arena, Allen & Ledgeway, 2012; Bogfjellmo, Bex & Falkenberg, 2013; Pilz, Miller & Agnew, 2017; Billino & Pilz, 2019). For example, performance has been shown to degrade with age in lower level, speed discrimination, and minimum motion tasks, as well as in higher-level motion coherence tasks (Snowden & Kavanagh, 2006). Additionally, Kavcic et al. (2011) found specific age-related deficits in heading and speed perception tasks using global optic flow stimuli. Further research has found differences in a motion-based spatial suppression task as a function of age (Betts et al., 2005; Tadin & Blake, 2005). In particular, Betts et al. (2005) demonstrated age-related differences in motion direction discrimination for large versus small high-contrast patterns in older participants compared to younger participants. Of particular relevance for the present study is that this result suggests potential age-related changes in local and global motion processing mechanisms, which would have implications for flow parsing. More generally, taken together, this body of research presents substantial evidence for specific age-related changes in both lower-level local, and higher-level global motion processing.

Although both non-visual and visual systems play a role in the detection/assessment of scene-relative object movement during self-movement, here we will focus on the purely visual optic flow parsing mechanism. To date, the extent to which this mechanism might change with age has been overlooked. Moreover, given the potential importance of its contribution to functional vision and the range of purely visual motion processing related deficits in older observers outlined above, it seems appropriate to test this. Based on previous research on visual motion processing, it would seem appropriate to predict a decline in the flow parsing mechanism with age. However, it has been suggested (Howard, Jenkin, & Hu, 2000) that greater emphasis is placed on visual processing in older age, because other nonvisual systems degrade more significantly. If this were true, then the effects of age might not be so marked. To examine these questions, we recruited 30 participants from a wide range of ages and conducted two flow parsing experiments to probe different aspects of flow parsing across the adult lifespan, as well as recording a range of neuropsychological measures.

In Experiment 1 we used a paradigm we have developed previously for measuring ability to detect scene-relative object movement from visual information alone (Rushton, Chen, et al., 2018). In this paradigm, stationary participants view a field of three-dimensional objects moving on the screen (and the retina). Crucially, the onscreen motion of the objects is consistent with the optic flow generated when an observer moves through a scene containing an array of scene-stationary items (see Figure 2). Participants must make judgements about the direction of movement of a probe object that has an additional scene-relative component of movement independent of the simulated observer movement. By manipulating the magnitude of the probe's scene-relative movement, it is possible to recover the observer's sensitivity to this information, i.e., a measure of the ability to detect scene relative movement amongst a complex background of optic flow. In recent work, we have provided evidence that ability to do this task is based on global motion information alone (i.e., optic flow subtraction), rather than other cues that might be available in the display (Rushton, Niehorster, Warren, & Li, 2018).

In Experiment 2 we examined the motion processing that underpins flow parsing directly, using a second task that measures the magnitude of global optic flow subtraction (Warren & Rushton, 2008; Warren & Rushton, 2009a; Warren et al., 2012; Foulkes, Rushton, & Warren, 2013a). In this paradigm participants view an expanding optic flow field consistent with forwards self-movement through a cloud of dots, together with a probe dot moving vertically upwards displaced either to the left or right of the fixation point (e.g., see Figure 6A). Participants report the perceived trajectory of the probe dot by adjusting the orientation of an on-screen paddle to represent the direction of perceived probe movement. Under flow parsing, subtraction of an expanding (i.e., outward) optic flow pattern leads to a perceived inward motion component added to the physical probe trajectory. The net effect is a perceived tilt (relative to the physical trajectory) toward the center of the radial flow field, which we refer to as the relative tilt. This effect is extremely robust, and, crucially, the effect persists even when optic flow is removed from the hemi-field containing the probe (Figure 6A). The relative tilt observed in this condition therefore provides a measure of the magnitude of flow parsed out by a global optic flow subtraction mechanism.

To preempt our results, in Experiment 1 we found evidence that the ability to detect object movement during self-movement using purely visual information does not change as a function of age, suggesting flow parsing is, on average, preserved for healthy older people. However, in Experiment 2, which we suggest measures characteristics of the underlying process that supports performance in Experiment 1, we found evidence that the magnitude of global optic flow subtracted increased significantly as a function of age. Together these results suggest age-related changes in the underlying processing that supports detection of object movement during self-movement and that these changes are sufficient to preserve performance in the face of other age-related motion processing deficits.

## General methods

### Observers

There is no existing data on flow parsing in older participants, but previous studies of global motion perception and ageing have demonstrated large effect sizes (Cohen's d) between 0.8 to 1.2 (Snowden & Kavanagh, 2006; Kavcic et al., 2011). Based on these results, power calculations (G*Power, Erdfelder, Faul, Buchner, & Lang, 2009) suggested a sample size of 30 was appropriate. Participants aged between 20 and 76 years with normal or corrected-to-normal vision and no history of neurological disorders were recruited through advertisements across the University of Manchester and public spaces across the city, as well as in the newsletters of local branches of the University of the Third Age (U3A). All participants took part in both tasks, i.e., Experiments 1 and 2 detailed below. The study was approved by the Ethics Panel of the Division of Neuroscience and Experimental Psychology on behalf of the University of Manchester's Research Ethics Committee, and all participants gave written informed consent.

### Cognitive/Visual testing

To ensure that participants in our wide age range sample were broadly similar in their cognitive function and to confirm that there was no pathological performance, all participants took part in a set of cognitive tests. Cognitive function tests were undertaken before the behavioral experiments and order was counterbalanced across participants. A summary of performance is provided in Table 1. It is worth emphasizing here that the primary purpose of these cognitive tests was to make sure our sample did not contain participants with atypical cognitive performance. As a consequence, with the exception of IQ, we do not refer to these metrics again in following sections. With respect to IQ, we were aware at the outset of previous studies (e.g. Melnick, Harrison, Park, Bennetto, & Tadin, 2013) providing robust evidence for a relationship between IQ and suppression of visual motion information in a spatial supression task. Given that flow parsing is also likely to invovle supression of motion (optic flow), there is theoretical motivation for considering the extent to which IQ might predict flow parsing measures in our experiments (see below).

**Table 1.** Demographics and cognitive test results. *Notes*: The normal range for AQT naming time is less than 60s for the color-form test and less than 50s for the color-number and -letter tests.

	Descriptive			Correlation		
	statistics			with age		
Variable	*n*	*M*	*SD*	*BF_10_*	*r*	*p*
Age						
All	30	49.23	19.03			
Female	19	50.26	19.03			
Male	11	47.46	19.82			
Cognitive function						
AQT naming time (s)	30					
Color-form		47.90	8.39	0.27^†^	−0.11	0.558
Color-number		42.00	6.22	0.23^†^	−0.01	0.960
Color-letter		43.67	6.83	0.54	0.25	0.180
WASI-II						
FSIQ-2	30	115.40	15.04	1.19	0.34	0.063

† Some evidence for no correlation with age (BF_10_ < 0.33).

Cognitive function was assessed using the AQT: A Quick Test of Cognitive Speed (Wiig, Nielsen, Minthon, & Warkentin, 2002) and the Wechsler Abbreviated Scale of Intelligence-Second Edition (WASI-II; Wechsler, 2011), which uses a subset of tests from the more comprehensive Weschler Adult Intelligence Scale-Fourth Edition (WAIS-IV; Wechsler, 2008) for quicker administration.

The AQT assesses automaticity, speed and working memory for processing visual stimuli, and for assessing general speed of cognitive processing. For this test participants completed all three primary tasks (A–C), which each comprised two single-dimension naming tests and one dual-dimension combination-naming test. For example, in A1 participants named the color of each item; in A2 participants named the form of each item; and in A3 participants named the color and form of each item. Naming time (seconds taken to read all items) and naming accuracy (number of errors) were recorded for all nine tests. In line with the test manual, naming-times for the three combination-naming tests (color-form, color-number and color-letter) were compared against criterions to determine within which range individual performance lay (normal, slower-than-normal, nonnormal, or pathological). These naming times are reported in Table 1. All but seven of our observers scored in the normal naming-time performance range (<60 seconds in color-form, <50 seconds in color-number and color-letter), with the remainder in the slower-than-normal range (i.e., no observers in the nonnormal or pathological ranges). In accordance with the AQT manual, slower-than-normal performance in these seven participants was subsequently investigated by considering naming accuracy. All participants scored in the normal naming-accuracy performance range (≤2 errors) suggesting that performance was not problematic. Consequently we suggest that AQT performance in our sample is broadly typical.

The Full-Scale IQ-2 Subtests (FSIQ-2) of the WASI-II, verbal Vocabulary and nonverbal Matrix Reasoning, were selected to obtain a general summary of intellectual ability (McCrimmon & Smith, 2013). For each participant we estimated IQ from a composite FSIQ-2 score (Table 1). All participants scored above 90 (Maccow, 2011), with a mean score around 115. These data suggest that our sample was not atypical with respect to IQ (Table 1).

Across all measures of cognitive function taken there were no significant correlations with age. Taken together with the general performance levels in the AQT and WASI-II tests outlined above this suggests that cognitive function was typical in our participants and was similar across the broad age range of our sample.

In addition to the tests of cognitive function, we administered a RANDOT test (Stereo Optical, Inc., Chicago, IL, USA), which provides a course, range-based measure of visual stereo-acuity. This was used simply to provide an exclusion criterion because performance in Experiment 1 relied on use of stereoscopic information. All participants exhibited some degree of stereo sensitivity (with all except four participants having stereoacuity at 70 arcsec or better), so no participants were excluded on these grounds (see Appendix for more information on RANDOT scores in our sample).

### Apparatus

Stimuli and tasks in both experiments were programmed using Lazarus, a public domain Pascal IDE. Experiments were run on a Windows 7 computer using an NVIDIA Quadro 600 graphics card and displayed on a 1680 × 1050 pixel (47.3 × 29.6 cm) Samsung LCD monitor at 120 Hz. In Experiment 1 stimuli were presented in stereo by temporally interleaving left and right frames and viewing through synchronized NVIDIA shutter glasses. In Experiment 2 stimuli were two dimensional and presented binocularly, in line with previous flow parsing studies (Warren & Rushton, 2009a; Warren & Rushton, 2009b). In both studies participants were stationary, seated in a dark room with their heads stabilized on a chinrest such that the eyes were approximately 57 cm from the screen. Forward observer-movement was simulated by presenting onscreen patterns of radial motion consistent with such a movement.

### Analysis

Where possible we have conducted Bayesian statistical analyses, since, in contrast with null hypothesis significance testing (NHST), they allow us to assess the balance of evidence supporting either the research or null hypotheses. We report Bayes factors (BF_10_—i.e., the relative strength of the evidence for the research hypothesis compared to the evidence for the null hypothesis) and interpretation of these quantities follows the convention outlined by Wagenmakers, Love, Marsman, Jamil, Ly, Verhagen, Selker, Gronau, Dropmann, Boutin, Meerhoff, Knight, Raj, van Kesteren, van Doorn, Šmíra, Epskamp, Etz, Matzke, de Jong, van den Bergh, Sarafoglou, Steingroever, Derks, Rouder & Morey (2018). All Bayesian analyses were performed using the JASP software package (JASP Team, 2020), using default priors. The default prior for Bayesian *t*-tests in JASP is described by a Cauchy distribution centered on 0, with a width parameter of 0.707. This corresponds to a probability of 80% that the effect size lies between −2 and 2. The default prior for Bayesian correlations is described by a beta-distribution centered around 0, with a width parameter of 1. This corresponds to a flat line, or continuous uniform distribution, and a probability of 80% that the correlation coefficient lies between −0.75 to 0.75. These are suitable ranges given effect sizes found in previous research (Tran et al., 1998; Snowden & Kavanagh, 2006; Kavcic et al., 2011; Bogfjellmo et al., 2013). For those readers who are more familiar with the NHST approach, we also report the outcome of more traditional Pearson's *r* and *t*-test analyses.

## Experiment 1

### Stimulus and procedure

**Figure 1.**
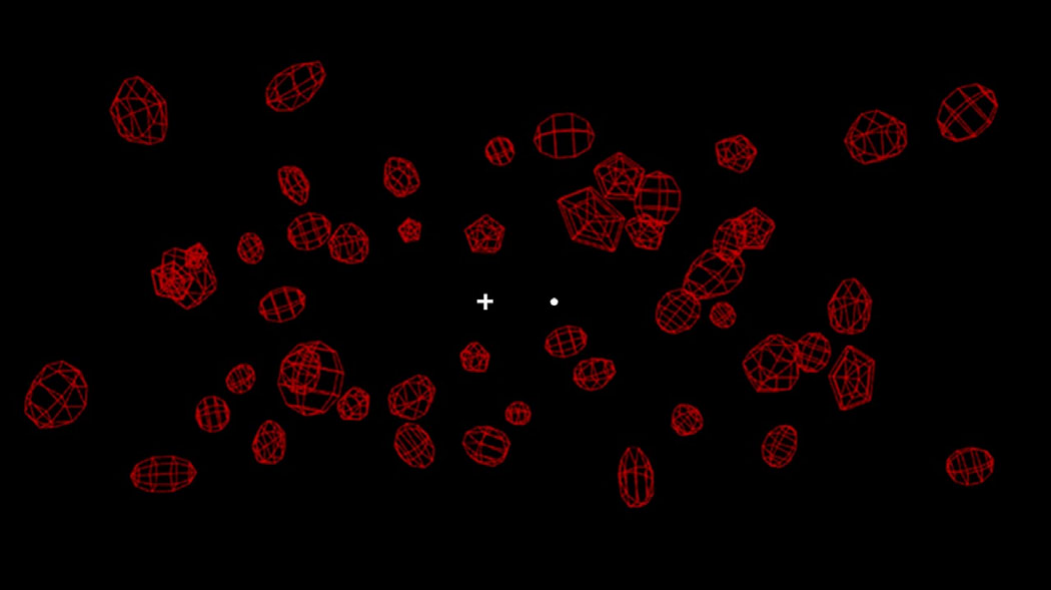
A single frame (monocular view) of an example stimulus from Experiment 1. Stimuli comprised a stereoscopically presented three-dimensional array of red wireframe background objects with a central fixation cross and probe dot to the right or left of fixation.

The stimulus in each trial consisted of 55 red wireframe background objects (see Figure 1) positioned within a volume of approximately 35 × 14 × 50 cm^3^ centered on a point 82cm from the observer. Shape locations were initially generated on a 11 × 5 regular grid (3.5 cm element separation) with origin at the center of the display and at 82 cm from the eye. These positions were then given small random jitter in the x and y direction by sampling from a uniform distribution over the range [−1.2, 1.2] cm. Element depth was assigned randomly in the range of −25 ≤ *z* ≤ 25 cm from the center of the array. When viewing stimuli through the NVIDIA stereoscopic shutter glasses the resulting percept was of three-dimensional objects floating at different depths in space (Figure 1). We chose to use close objects to minimize the conflict between accommodation and convergence associated with viewing a stereo display (see Wann, Rushton & Mon-Williams, 1995). So that angular velocities remained comparable to those experienced when walking, simulated self-movement speed was scaled down accordingly (see Rushton, Chen & Li, 2018, for further explanation). A white fixation cross was presented at the center of the volume and a white probe sphere (0.2° diameter) was randomly presented either −2.4° (left) or 2.4° (right) from the fixation cross at the same depth. After pilot testing, we ascertained that we could minimize the chances of the probe and background objects intersecting by removing objects that were initially in a 10° × 2° range surrounding the fixation cross. In the unlikely event that intersecting did occur, the probe would never be fully occluded since the background objects were wireframe. The image of the scene was initially static for one second to allow the observer time to fuse the stereoscopic images, before the trial motion was presented for an additional one second. Throughout the trial participants were asked to fixate on the cross while they judged the perceived direction of movement (left or right) of the probe relative to the scene (i.e., the background objects). After an initial series of practice trials, participants had no difficulty completing this task.

**Figure 2.**
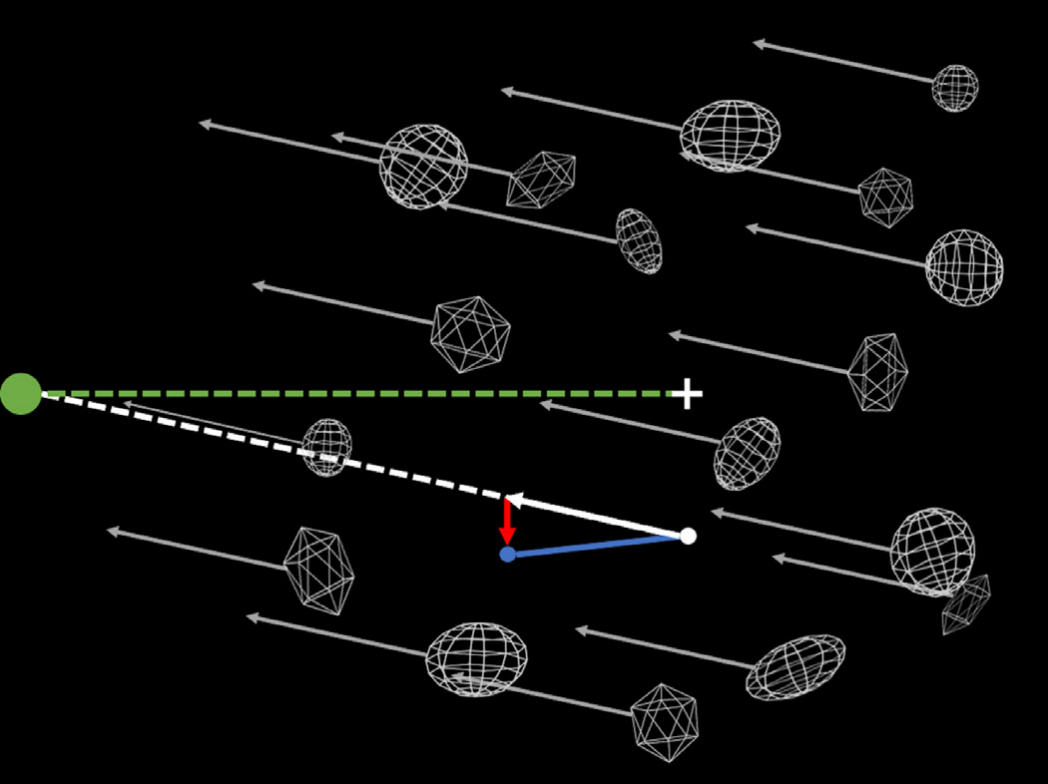
Simplified birds-eye view of the stimulus in Experiment 1 (not to scale). Green sphere indicates the position of the observer relative to the stimulus (not to scale). The green dotted line represents the direction of the observer's gaze to the white central fixation cross and therefore the center of the field of view. The gray arrows indicate the direction of movement of the scene toward the observer. The white sphere indicates the initial position of the probe dot at the same depth as the fixation cross. The probe moves with the scene (white arrow) but with an added rightward or leftward (depending on the trial) scene-relative motion (red line). The resulting motion of the probe (blue sphere) is indicated by the blue line.

Two experimental conditions were investigated. These conditions were blocked, and order was counterbalanced across participants. In the stationary condition the background objects were static throughout the trial, consistent with the observer being stationary. In the moving condition (see Figure 2) all elements in the scene except the fixation cross (i.e., the background objects and the probe) were given an optic flow component consistent with forward observer movement at 12.5 cm/s toward the initial position of the probe (i.e. ±2.4° from the fixation cross). In both moving and static conditions, the probe was given an additional purely horizontal component of motion, which could be either to the left or right relative to the scene. After the trial ended a black screen was presented and the participant was asked to report the perceived scene relative movement (left or right) of the probe via a mouse button press.

Scene-relative lateral probe movement was controlled on each trial via a Kesten staircase (see Treutwein & Strasburger, 1999). This staircase changes step size on each trial depending on the number of previous reversals and enables convergence to a pre-defined point on a psychometric function. Two such staircases were interleaved for each of the static and moving conditions. One staircase started with strong rightward probe motion 1 cm/s (+ve is rightward in our sign convention) and velocity was reduced (by initial step size of 0.33cm/s) if participants made rightward responses and could eventually change sign (to leftward motion). This staircase was set to converge to a probe velocity at which point 20% of responses were rightward. The other staircase worked in the opposite direction starting with strong leftward probe motion (−1 cm/s) and was set to converge to a probe velocity at which point 80% of responses were rightward. Using these start values and points of convergence on the psychometric function meant that our staircases crossed, thereby ensuring good coverage of data points around the critical central region of the psychometric function.

Participants completed two sessions with a short break between. For both sessions and both conditions, each of the two staircases had 50 trials. In practice this was easily sufficient for staircase convergence with the vast majority of staircases converging within 30 trials. Individual psychometric functions for each participant and in each condition were therefore generated based on 200 trials (50 trials × 2 staircases × 2 sessions). Participants first undertook several practice trials that were later discarded from the analysis.

### Analysis

**Figure 3.**
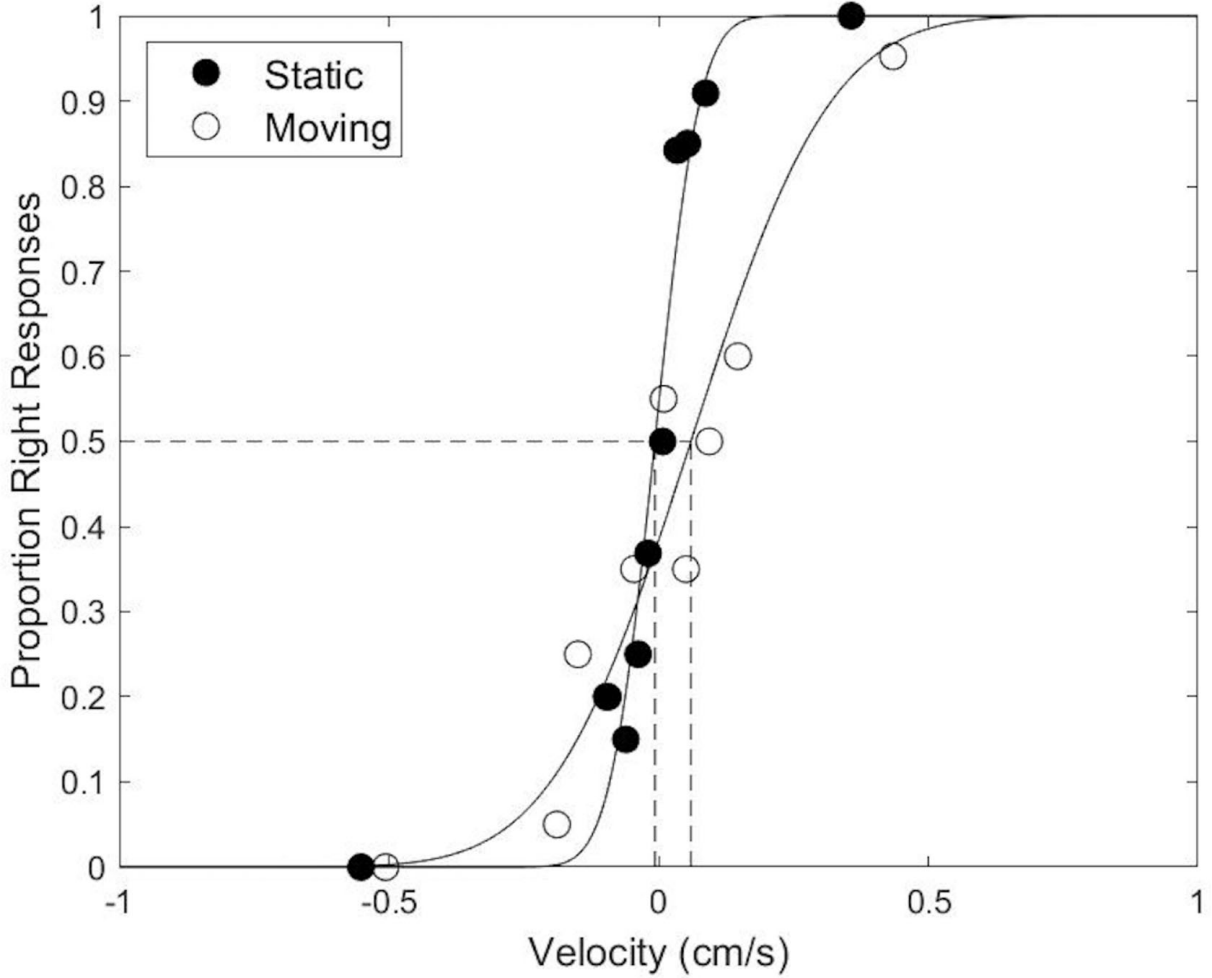
Example psychometric functions from one participant for the static and the moving conditions. Psychometric functions were fitted to the binary response data, while estimated proportion of rightward responses, aggregated over similar velocities, are also shown for illustrative purposes only (filled markers: static condition; unfilled markers: moving condition). Vertical dotted lines indicate the point of subjective equality (PSE) from the psychometric functions fitted to each condition.

Responses (left or right) were recorded for each trial. As no difference was expected between left and right probe presentation, data were collapsed over this manipulation in our analysis. Specifically, for conditions where the probe was on the left, responses were flipped and velocity was multiplied by −1. For each participant, in each condition, we fitted a cumulative Gaussian psychometric function to the binary response data using a maximum likelihood estimation procedure from the Palamedes toolbox (Prins & Kingdom, 2018; analysis code is provided at GitHub: *https://github.com/lucyevansUoM/flowparsing-ageing*). In Figure 3 we show two such example psychometric functions relating the scene-relative horizontal probe velocity to the probability of a rightward response fitted to the binary response data from a single observer in the static and moving conditions. For illustration purposes only we also show the estimated proportions of rightward responses for a range of velocities (open and filled circles). These were estimated by aggregating responses over similar velocities.

The mean and standard deviation of the fitted cumulative Gaussian functions were extracted for each participant. The mean encodes the point of subjective equality (PSE), i.e. the probe velocity that lead to 50% of rightward responses, i.e., at which the participant perceived the probe to be scene stationary. The standard deviation of the fitted psychometric function encodes the direction discrimination threshold for each participant. This threshold represents the precision with which the participant can discriminate left from right displacements relative to the scene. This measure is particularly relevant to our research question since it encodes sensitivity to scene relative probe motion. We expected this threshold to be worse (i.e., higher threshold or equivalently lower slope) in the moving condition, because scene-relative motion needs to be parsed to do the task in this condition (note this is consistent with the illustrative fits presented in Figure 3).

Crucially, we used the ratio of the moving condition threshold over the static condition threshold as our measure of a participant's ability to judge object movement during simulated self-movement. Doing so controls for individual differences between participants, since detection of object movement during self-movement is measured relative to an individual's direction discrimination threshold in the stationary condition. We predicted ratios greater than 1 because they indicate greater thresholds in the moving compared to the static condition. Ratios closer to 1 indicate a smaller difference between static and moving conditions, suggesting a greater ability to detect object movement during self-movement. We therefore report the flow parsing index (FPI) as the ratio between the moving and the static thresholds and correlate this measure with age to investigate the potential age-related effects.

### Results

**Figure 4.**
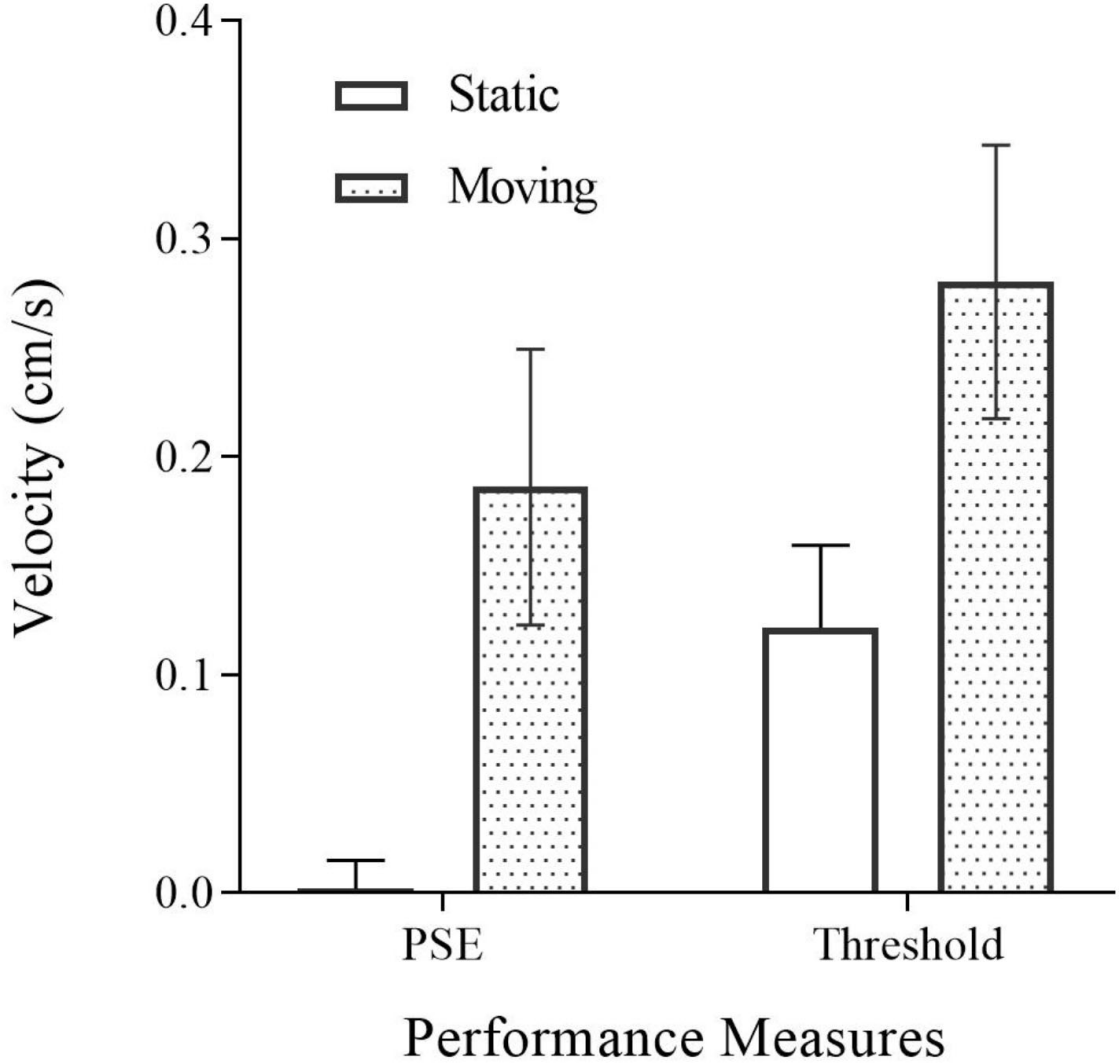
Mean group PSE and direction discrimination thresholds plotted for each condition; static (no fill) and moving (spotted). Error bars are 95% confidence intervals.

As anticipated, discrimination thresholds for the direction of scene-relative probe movement were larger for the moving than the static condition (Figure 4). The *t*-tests provided evidence that mean moving discrimination threshold was significantly higher than mean static discrimination threshold (*BF_10_* > 100, *t* = 6.06, *p* < .001). Nonetheless, the thresholds are still relatively small in the moving condition (<0.3 cm/s), and participants did not report any problems carrying out the task in this condition.

Although it does not relate to our research question on changes in ability to detect scene relative movement with age, for completeness, we also present the mean (over age) PSEs and thresholds for both the static and moving conditions. The PSE in the static condition should be close to zero, indicating that participants perceived the probe as stationary when it was not moving onscreen. This was supported by a one-sample *t*-tests providing evidence that static PSE was not different from 0 (*BF_10_* = .20, *t* = 0.31, *p* = 0.761). If the flow were fully parsed in the moving condition, then the PSE should also be close to zero (because then the moving and static conditions would be equivalent after parsing). There was clear evidence, however, that moving PSE was different from 0 (*BF_10_* > 100, *t* = 6.01, *p* < 0.001) suggesting a significant but small positive bias (0.186 cm/s). This indicates that when the probe was actually stationary it was perceived as moving slowly to the left in the scene. The reason for this small bias cannot be established from our data; it could arise because parsing is incomplete or because parsing is complete but inaccurate because of underestimation of object distance or overestimation of self-movement speed (or some combination of these factors).

**Figure 5.**
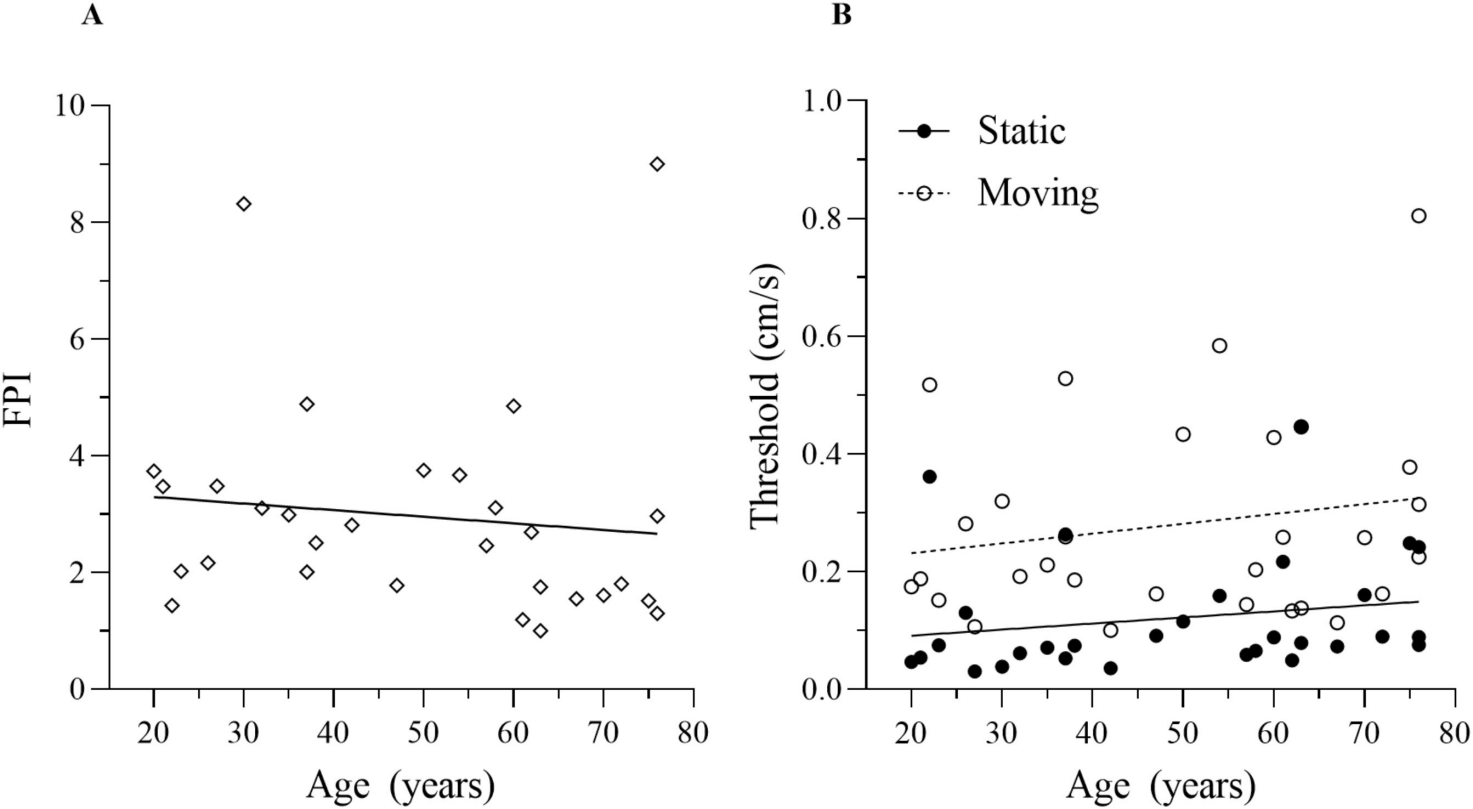
(A) The FPI, i.e., the threshold ratio (moving/static) plotted against age, with associated regression line (*r^2^* = 0.013). (B) Discrimination thresholds in the static (*r^2^* = 0.038) and moving (*r^2^* = 0.036) conditions plotted against age, with associated regression lines.

Of more relevance to our research question is the relationship between thresholds and age. Figure 5A shows the FPI (i.e., the ratio of moving to static thresholds) as a function of age. Note that threshold ratios (FPIs) are, as expected, typically greater than 1, mirroring the data presented in Figure 3 and suggesting that thresholds were higher in the moving than the static condition. Crucially, there does not seem to be a clear relationship in Figure 5A between FPI and age (*BF_10_* = 0.27, *r* = −0.12, *p* = 0.543), and this was also true when we correlated the simple difference in thresholds with age (*BF_10_* = 0.25, *r* = 0.08, *p* = 0.657). In fact the Bayes factors recovered suggest that there is some evidence for an absence of correlation between these factors. There was also little evidence of a correlation between static threshold (*BF_10_* = 0.38, *r* = 0.19, *p* = 0.304) or moving threshold (*BF_10_* = 0.37, *r* = 0.19, *p* = 0.317) and age separately (Figure 5B). Finally, unlike previous studies on suppression of motion information (which we assume is at play in flow parsing), we did not find a relationship between IQ and FPI in this experiment (*BF_10_* = 0.42, *r* = −0.21, *p* = 0.256).

### Discussion

In Experiment 1, we investigated ability to detect scene relative object movement during self-movement, using purely visual information and changes in this ability with age. As expected, there was a clear increase in direction discrimination thresholds (lower slope of psychometric function) in the moving relative to static condition across observers; however, the extent of this difference did not depend on age (Figure 5). Ability to detect scene relative object movement is therefore preserved across our broad age range sample and, more generally, might be preserved across the adult lifespan in the population. If this were the case then it would be noteworthy, given the marked changes observed in other local and global motion-based tasks with ageing. We propose that the ability to undertake this task relies primarily on optic flow parsing (Rushton, Niehorster, et al., 2018). If flow parsing is indeed preserved with age, then it is of interest to see whether the global subtraction process that underpins flow parsing is altered, and we address this question directly in Experiment 2.

## Experiment 2

### Stimulus and procedure

**Figure 6.**
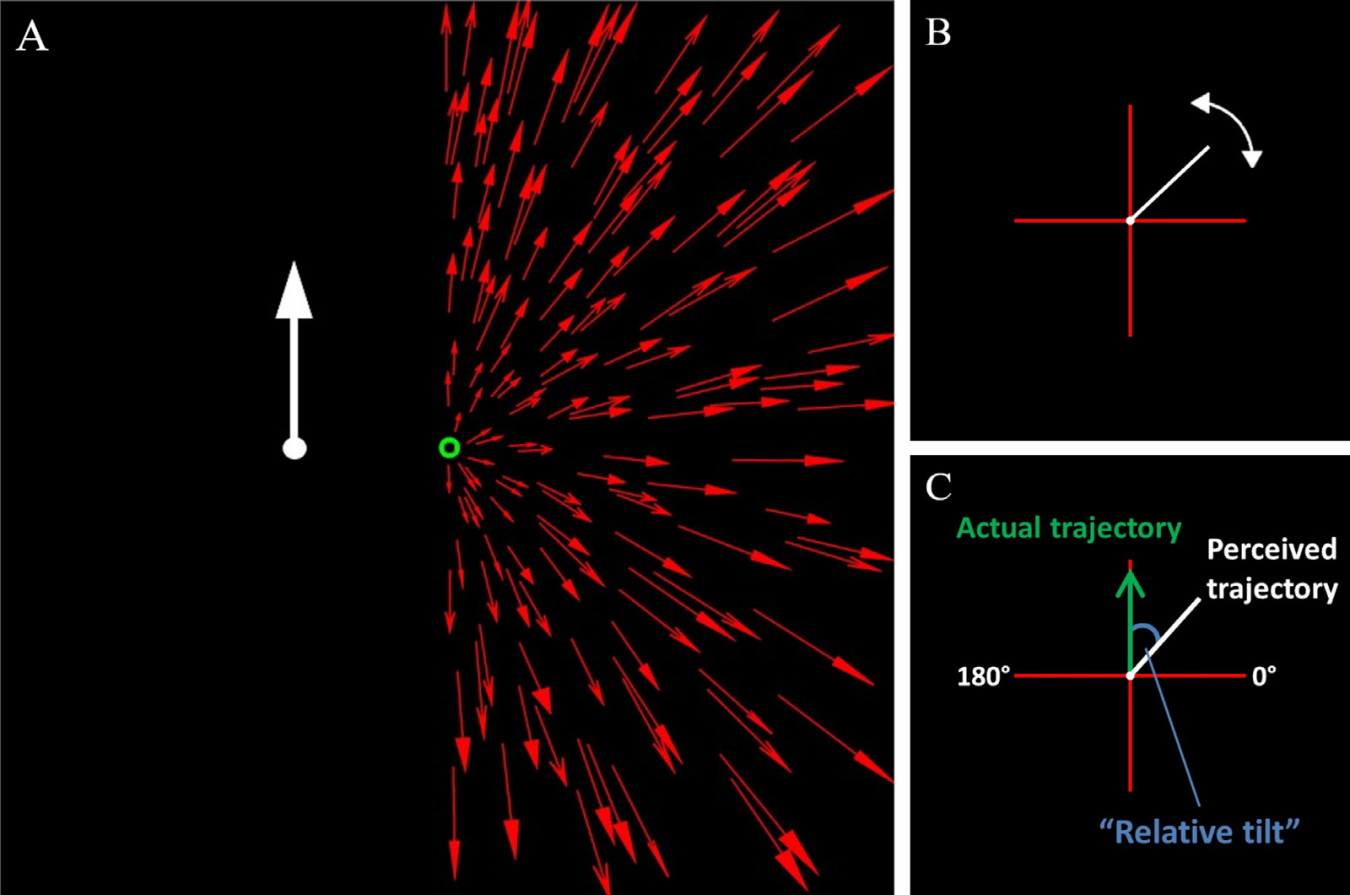
(A) Schematic of one example stimulus used in Experiment 2 with flow in the right hemifield and probe in the left hemifield. The central arrow represents the 90° condition in which the probe moved vertically upwards. (B) Schematic of the on-screen axis and paddle. Participants rotated the white line CW or ACW (between 0° & 180°) to represent the perceived trajectory of the probe. (C) Illustration of the relative tilt as the angular difference between the perceived and actual trajectories. Adapted from Warren & Rushton, 2009a.

Our methods were similar to several previous studies using a relative tilt task to probe optic flow parsing (e.g., Warren & Rushton, 2008; Warren & Rushton, 2009a; Foulkes et al., 2013a). The stimulus comprised an expanding limited lifetime optic flow field, generated as a virtual cloud of 300 red dots on a black background. Random uniform onscreen two-dimensional dot locations spanning the full screen size (47.3° × 29.6°) were generated before assigning the dots random depth between 107 cm and 207 cm (57 cm viewing distance + a uniform sample in the range [50, 150] cm) from the observer. The dot speed was consistent with simulated forward observer motion of 120 cm/s (i.e., a slow walking speed). Dots had a limited lifetime of 20 frames (166.67 ms), after which they were regenerated at a new random location to maintain dot density. In addition to the flow field, a probe dot was presented either −4° (left) or +4° (right) of a central fixation dot at the focus of expansion of the flow field. On each trial the probe moved at a speed of 0.8cm/s and at an angle of 75°, 90°, or 105° (measured clockwise relative to 0°, which was defined as rightward horizontal movement, Figure 6C). These angles were selected randomly on each trial so physical trajectories could not be predicted.

To enable us to consider the relative contributions of local and global motion processing mechanisms to the relative tilt effect we used three different flow fields: Full – flow field was present across the whole field (47.3° × 29.6°); HemiL – flow field was present only in the left hemi-field; HemiR – flow field was present only in the right hemi-field (see Figure 6A). Factorial combination of these three flow fields with the different probe position enabled us to contrast trials on which the probe and flow were present in the same versus opposite hemifields and therefore isolate the contribution of local and global motion processing mechanisms (see analysis section below).

Each 2.5-second trial consisted of 0.5-second presentation of the fixation cross alone, followed by 2-second presentation of the fixation cross together with the flow and probe stimuli. After each trial, observers were instructed to indicate the perceived trajectory of the probe by moving, with the computer mouse, an onscreen paddle, which could be moved freely to represent any angle between 0° and 180° (Figure 6B).

Participants completed two sessions of 108 trials per session made up of 3 (probe angles) × 3 (flow fields) × 2 (probe positions) × 6 (repetitions per condition) trials, so we had a total of 216 settings per participant.

### Analysis

Relative tilt (RelT) was calculated on each trial and for each participant as the angular difference between actual and perceived trajectories of the probe. In order to collapse over the three probe direction conditions, RelTs in the 75° and 105° conditions were transformed into an equivalent horizontal illusory component of motion at 90°, before being converted back to an angular quantity (see Equations 1 and 2 from Warren and Rushton, 2008 for details of this transformation). Consequently, after this process it was as if all data had been collected in the 90° probe direction, and so we collapsed subsequent RelT values over the probe direction conditions.

In line with Warren and Rushton (2009a) and to allow examination of local and global motion processing contributions to RelT, we recoded the six (probe position x flow field) conditions into six new (probe position x configuration) conditions. The configuration factor levels were: Full – flow was present in the full field, i.e. the (Field = Full, Probe = Left) and (Field = Full, Probe = Right) conditions; Same – flow was present only in the same hemifield as the probe, i.e., the (Field = HemiL, Probe = Left) and (Field = HemiR, Probe = Right) conditions; Opposite – flow was present only in the opposite hemifield to the probe, i.e. the (Field = HemiR, Probe = Left) and (Field = HemiL, Probe = Right) conditions. We did not predict an effect of probe position, so data were also collapsed over left and right probe presentation by flipping the sign of the left probe position data. Consequently, all data are reported as if the probe was on the right of the field for the three remaining conditions of the configuration factor (Full, Same, Opposite). An average RelT for each configuration condition was then calculated for each participant. Specifically, for each participant and condition this was based on the settings from 72 trials—i.e., 6 (repetitions) × 3 (probe orientations) × 2 (probe positions) × 2 (sessions). Unfortunately, two participants were unable to complete the second session of the experiment, so their RelTs were calculated from only 36 trials.

Consistent with previous studies (Warren & Rushton, 2009a), data in the different flow field conditions (Full, Same, Opposite) were used to estimate contributions of local and global motion processing mechanisms to the relative tilt effect. In the Opposite condition there was no (or minimal) motion information in the neighborhood of the probe. As a consequence, RelT observed in this condition must be due to global motion processing. In contrast, in both the Full and Same conditions there is motion near to the probe and RelT observed in these conditions is therefore due to a combination of both global and local motion processing. We assume that the former component reflects global subtraction of optic flow under flow parsing and suggest that the local component is driven by lower level center-surround motion contrast responses in early visual areas (e.g., see Frost & Nakayama, 1983). Since the contribution of global flow subtraction is present in both Same and Opposite conditions but that of local motion processing is only present in the Same condition, we are able to estimate the local contribution in isolation as the difference between RelT (Same − Opposite) in these conditions. We present the Full field data in the results section for completeness only. Any differences between RelT in the Full and Same conditions are likely driven by having more optic flow present in the Full condition.

### Results

**Figure 7.**
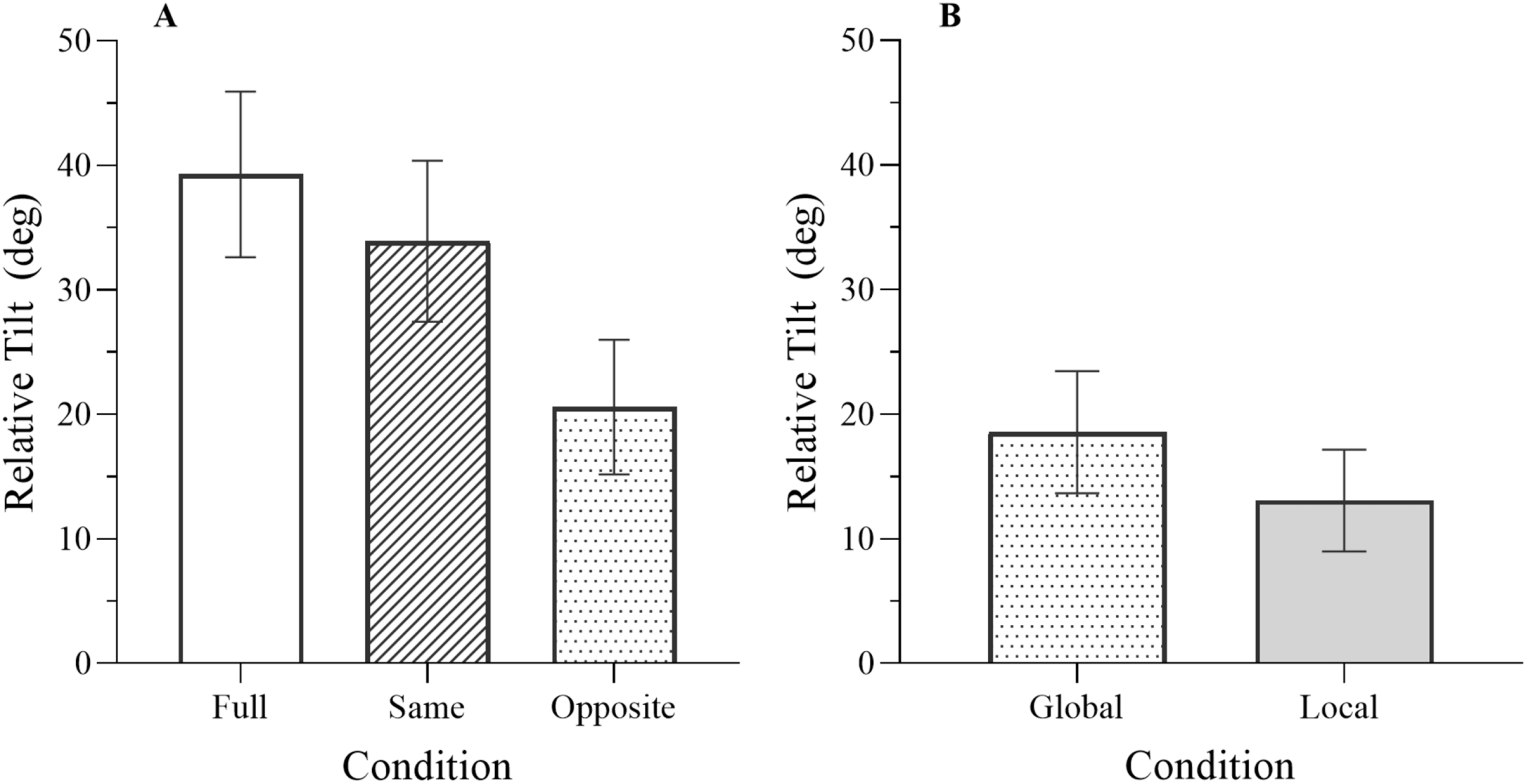
(A) Average relative tilt for the three conditions; full (unfilled), same (striped), and opposite (spotted). (B) Average local (filled) and global (spotted) relative tilts. Error bars are 95% confidence intervals.

Figure 7 shows the average relative tilt data for the Full, Same, and Opposite conditions (Figure 7A), together with the associated contributions of global (i.e., subtraction of optic flow) and local motion processing mechanisms (Figure 7B; note that the global contribution is simply the data from the Opposite condition whereas the local contribution is obtained as the difference between the Same and Opposite conditions as described above). In line with previous studies (Warren & Rushton, 2009a; Warren et al., 2012), relative tilts were largest in the Full and Same conditions, they were smaller in the Opposite condition and the tilt observed in the Opposite condition was around half the size of that observed in the Full and Same conditions (Figure 7A). In addition, the contribution of the global optic flow parsing mechanism was larger than that of local motion processing mechanisms (Figure 7B).

Using Bayesian one-sample *t*-tests there was strong evidence for the presence of a non-zero RelT effect for each of the Full (*BF_10_* > 100, *t* = 12.09, *p* < 0.001), Same (*BF_10_* > 100, *t* = 10.72, *p* < 0.001), and Opposite (*BF_10_* > 100, *t* = 7.77, *p* < 0.001) conditions. Similarly, there was strong evidence for the presence of a non-zero local motion processing contribution to RelT in the same condition (*BF_10_* > 100, *t* = 6.99, *p* < 0.001). Paired Bayesian *t*-tests suggest that mean relative tilt in the Opposite condition was significantly less than mean Full relative tilt (*BF_10_* > 100, *t* = −9.18, *p* < 0.001) and mean Same relative tilt (*BF_10_* > 100, *t* = −6.99, *p* < 0.001). There was also strong support for a difference between RelT in the Same and Full conditions (*BF_10_* > 100, *t* = −9.55, *p* < 0.001) suggesting that the presence of more (or at least more robust) flow information led to slightly higher RelT effect. There was much weaker evidence for a difference in the contribution of global and local mechanisms to the RelT effect (Figure 7B) (*BF_10_* = 1.47, *t* = 2.17, *p* = 0.039).

**Figure 8.**
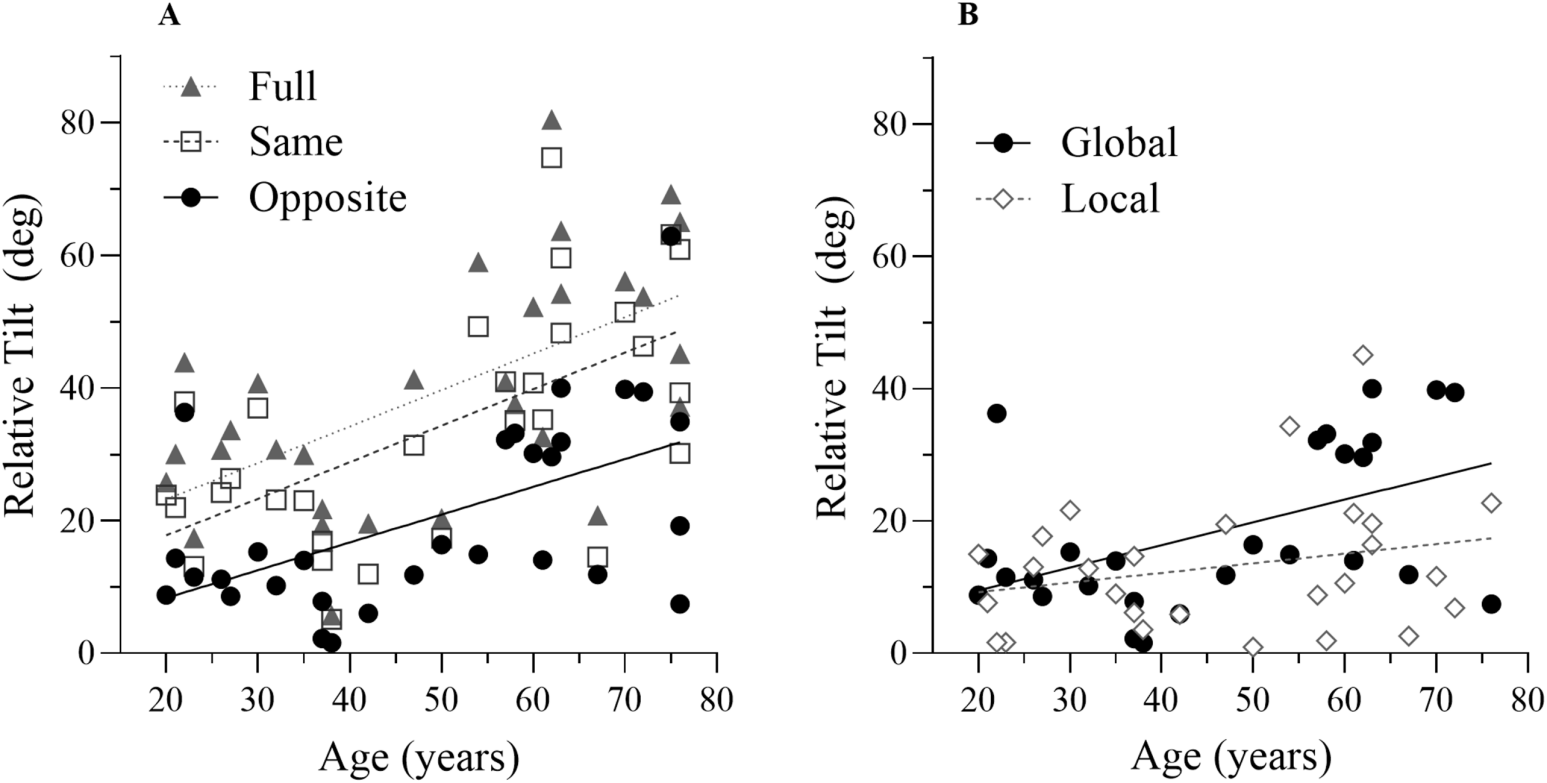
(A) Full (filled triangle; *r^2^* = 0.346), Same (square; *r^2^* = 0.367), and Opposite (filled circle; *r*^2^ = 0.303) relative tilts plotted against age, with associated regression lines. (B) Global (filled circle; *r*^2^ = 0.303) and Local (diamond; *r*^2^ = 0.058) contributions to the relative tilt effect as a function of age, with associated regression lines.

Turning to our primary research question about changes in flow parsing with age, Figure 8A suggests there is a positive correlation between RelT and age in all conditions, with older participants perceiving a larger deflection of the probe than younger participants. When local and global contributions to RelT are plotted against age (Figure 8B), it appears that there is a positive relationship between age and Global RelT, but not age and Local RelT. This suggests that the change in RelT with age is driven primarily by the global subtraction of optic flow. Bayesian and Pearson correlations were conducted to test these observations formally. They indicated very strong evidence for relationships between age and Full relative tilt (*BF_10_* = 59.41, *r* = 0.59, *p* < 0.001), and Same relative tilt (*BF_10_* = 92.15, *r* = 0.61, *p* < 0.001), and a strong relationship between age and Opposite (and equivalently global) relative tilt (*BF_10_* = 25.64, *r* = 0.55, *p* < 0.002). In contrast, the estimated Local relative tilt showed no clear evidence of a correlation with age (*BF_10_* = 0.50, *r* = 0.24, *p* = 0.198), suggesting this relationship was predominantly between age and the Global motion processing contribution to RelT.

### IQ as a predictor of relative tilt?

As noted above, previous research has suggested a strong link between IQ and performance in a task probing suppression of motion information (Melnick et al., 2013). Similarly, we found evidence that the IQ measure used in the cognitive testing battery (FSIQ2) correlated with relative tilt in all conditions (Same: *BF_10_* = 27.33, *r* = 0.553, *p* = 0.002; Opposite: *BF_10_* = 11.84, *r* = 0.510, *p* = 0.004; Full: *BF_10_* = 19.57, *r* = 0.537, *p* = .002). To examine the potential issue that IQ (and not age) was the primary predictor of RelT we conducted a two-step hierarchical regression analysis to investigate whether FSIQ2 could have accounted for the age-related change in Global (Opposite) RelT observed. The regression statistics are reported in Table 2. In the first step, age was added to the model and was found to contribute significantly (*F*_(1,28)_ = 12.14, *p* = 0.002), accounting for 30.2% of the variation in Global relative tilt. In step two, introducing FSIQ2 to the model explained only an additional 11.7% of variation in Global RelT although this change in *R^2^* was significant (*F*_(1,27)_ = 5.43, *p* = 0.028). Although FSIQ2 therefore contributed to the variation in Global RelT, the most important predictor was age, which uniquely explained 30.2%. Adding the factors in the opposite order produced similar effects.

**Table 2.** Summary of hierarchical regression analysis for variables predicting Global RelT. *Notes*: N = 30; * *p* < 0.05, ** *p* < 0.01, *** *p* < 0.001.

	*R*	*R^2^*	*∆R^2^*	*B*	SE *B*	*β*
Step 1	0.55	0.30	0.30**			
Age				0.42	0.12	0.55**
Step 2	0.65	0.42	0.12*			
Age				0.32	0.12	0.43*
FSIQ-2				0.35	0.15	0.36*

### Discussion

In this experiment we investigated the relationship between age and the global flow subtraction process that underpins flow parsing, using a relative tilt task similar to that in Warren and Rushton (2009a). We found RelTs of a round 35° to 40°in the Full and Same conditions and around 20° in the Opposite condition, consistent with previous data (e.g., Warren & Rushton, 2009a). We also found evidence for a positive correlation between age and RelT in the Opposite (and equivalently Global) condition, suggesting that the component of optic flow that is globally subtracted under flow parsing increases with age.

## General discussion

### Summary

In two experiments we investigated aspects of the optic flow parsing mechanism across a wide age range. In Experiment 1 we measured sensitivity to scene-relative object movement during self-movement on the basis of visual cues alone. We found that this sensitivity was largely preserved with age. Crucially, ability to perform this task is underpinned by the subtraction of global optic flow in flow parsing (Rushton, Niehorster, et al., 2018). In Experiment 2 we looked for age-related changes in this global subtraction process and found that relative tilt effects driven by global subtraction of optic flow increase with age.

### Experiment 1: Preserved detection of scene relative movement with age

The finding that sensitivity to (and thus the ability to detect) scene-relative object movement during visually simulated self-movement was preserved with age is, on first inspection, surprising considering the range of motion processing deficits seen previously for older adults in both lower and higher level motion processing tasks (e.g., Snowden & Kavanagh, 2006; Kavcic et al., 2011). Snowden and Kavanagh (2006) reported significantly impaired performance in both speed discrimination and minimum motion detection tasks. They also found evidence for impaired performance in a higher-level motion coherence task that involves integrating motion over the visual field (similar to optic flow processing). Perhaps more surprising in light of the present results are the data of Kavcic et al. (2011), who found significantly impaired heading discrimination thresholds in older participants. Given that both heading recovery (Lappe, Bremmer & van den Berg, 1999) and flow parsing are thought to depend on optic flow processing, these findings raise the question of why we did not observe similar impairments with age in Experiment 1. Of relevance here are our previous studies suggesting flow parsing and heading rely on different processing. First, flow parsing does not depend on prior recovery of heading (Warren et al., 2012). Second, the precision of flow parsing is not limited by the precision of heading recovery (Rushton, Chen, et al., 2018). Experiment 1 therefore reinforces the idea that heading and flow parsing, despite both relying on optic flow, involve different neural processing.

Based on the results of Experiment 2, we propose that the primary reason for preserved performance in Experiment 1 is that the underlying neural systems supporting detection of object movement change with age. Moreover, we suggest that this might occur to counteract other motion processing deficits experienced in ageing. If such changes occur in the underlying neural processing, then this may reflect the functional importance of detecting scene relative object movement during self-movement. This would not be surprising since impairments in detection and assessment of scene-relative movement could lead to problems with avoiding obstacles and, ultimately, have consequences for health and safety (e.g., greater incidence of collisions). Note that although average performance in Experiment 1 was preserved with age there was more variability in discrimination thresholds in older participants. Those older individuals for whom performance is not preserved may then be vulnerable to increased collision or fall risk for this reason.

### Experiment 2: Increased optic flow subtraction with ageing

The positive correlation between the relative tilt measure and age in the Opposite (and equivalently Global) condition indicates an increased magnitude of global flow subtraction in older observers. On first glance this might seem out of line with the prior literature given that, as mentioned above, many previous studies on motion processing have reported evidence of impairment in ageing observers (Tran, Silverman, Zimmerman & Feldon, 1998; Betts, Taylor, Sekuler and Bennett, 2005; Tadin & Blake, 2005; Snowden & Kavanagh, 2006; Bennett, Sekuler & Sekular, 2007; Billino, Bremmer & Gegenfurtner, 2008; Kavcic, Vaughn & Duffy, 2011; Hutchinson, Arena, Allen & Ledgeway, 2012; Bogfjellmo, Bex & Falkenberg, 2013; Pilz, Miller & Agnew, 2017; Billino & Pilz, 2019). However, the study conducted by Betts et al. (2005) has interesting parallels with our results. Betts et al. (2005) used a spatial suppression task to measure the threshold stimulus duration for which participants could reliably discriminate the direction (left vs. right) of high-contrast drifting gratings of different sizes. In younger observers, duration thresholds increased with stimulus size, whereas in older participants the change in threshold with size was less marked. In fact, older participants required less time than younger observers to reliably discriminate direction of motion for the largest stimuli tested. The explanation offered was that the worse performance in younger relative to older observers arose because larger stimuli incur greater suppression from the surround of center-surround cells. The apparently better performance in older observers for large stimuli would then arise if there were an age-related deficit in surround suppression, which has been suggested by several authors (Betts, Sekuler & Bennett, 2009; Karas & McKendrick, 2012; Deng, Chen, Kuang, & Zhang, 2017) and is thought to rest on reduced GABA-mediated inhibition in the ageing visual system (Gao, Edden, Li, Puts, Wang, Liu, Zhao, Wang, Bai, Zhao, Wang, Barker, 2013; Leventhal, Wang, Pu, Zhou, & Ma, 2003). Alternatively, the apparent performance enhancement might arise from increased central summation to compensate for reduced signal to noise ratios observed in older primate visual cortex (Schmolesky, Wang, Pu & Leventhal, 2000). Under the account proposed in Betts et al. (2005), the change with increasing age has negative functional consequences because spatial suppression segments smaller regions of relative motion from larger surround motion likely due to observer movement (a role that sounds strikingly similar to that of optic flow parsing). In summary, Betts et al. (2005) provide a prior example of how performance on a large field motion task can actually improve with age, although it is suggested that this is associated with worse functional vision.

The question remains then of why, if visual motion processing is generally impaired in older participants, is there greater subtraction of optic flow in this group? Work by Howard et al. (2000) on the perception of body orientation may provide an answer. Howard et al. (2000) found that older participants have greater reliance on retinal cues to body orientation than extra-retinal cues. They suggest that this is because sensitivity to extra-retinal cues (e.g. vestibular information) declines more rapidly than sensitivity to retinal cues and therefore the latter information sources are up-weighted. A similar process may underlie the effects observed in Experiment 2, i.e. increased subtraction of global optic flow may reflect a reweighting of retinal and extra-retinal cues to self-movement in favor of retinal information.

Also consistent with the reweighting explanation are recent findings on changes in induced motion in older observers (de Dieuleveult, Brouwer, Siemonsma, van Erp & Brenner, 2018; de Dieuleveult, Perry, Siemonsma, Brouwer & van Erp, 2019). Induced motion is commonly experienced as illusory movement of an object in the opposite direction to the movement of its surrounding background (Duncker, 1929). Dieuleveult et al. (2018) argued that the stronger influence of the background motion in older participants was due to lower sensitivity in proprioceptive and vestibular systems and thus greater dependence on visual information.

### IQ and suppression of optic flow

As part of our analysis to confirm that the observed age effects were not driven by differences in IQ, we assessed the correlation between IQ and the magnitude of optic flow subtraction. Higher IQ correlated with RelTs in all three flow field conditions in Experiment 2, although we confirmed that age was the primary predictor of these effects. Previous studies have linked IQ to performance in motion perception and psychophysical studies and, in particular, for the spatial suppression task discussed above (Melnick et al., 2013; Tadin, 2015). Melnick et al. (2013) linked high intelligence with greater ability to suppress irrelevant or background information and suggested that this was required both in IQ tests and in perceiving motion relative to an irrelevant background. The data presented here are, at least, consistent with this proposal, where the background information to be suppressed is an optic flow field. Clearly such visual information is not irrelevant because it contains useful information about observer movement. However, in the context of recovering the scene-relative movement of other parts of the environment, one might argue optic flow is a background signal to be suppressed since it makes this task harder.

### Potential limitations

It is, of course, possible that a more general visual issue in older participants drives the changes in relative tilt observed in Experiment 2. For example, as noted in Appendix, there is some evidence for a positive correlation between RANDOT scores and age in our group. However, note that there does not appear to be a relationship between RANDOT and either FPI or RelT. Alternatively, conditions such as presbyopia are generally more common in older people. It is not obvious, however, how these differences might lead to a systematic effect of increased relative tilt with age. Such an effect would occur if objects in the scene were perceived to be closer (requiring a larger optic flow component to be subtracted). However, to generate the significant (approximately threefold) increase in relative tilt observed here, the perceived change in distance to the probe would need to be unrealistically large. Consequently, even if such factors contribute in small part, it is unlikely that they could completely explain the effects observed in Experiment 2.

A potential issue with Experiment 1 is that participants did not follow instructions and, rather than making scene-relative judgements, reported probe movement relative to (e.g.) the edge of the screen, fixation point or even relative to themselves. Given our stimulus these responses would be indistinguishable. However, we suggest this is unlikely for a number of reasons: (1) participants performed practice trials before embarking on the task and were regularly reminded about the task instructions throughout; (2) it is likely to be easier or just as easy to report movement relative to the scene surrounding the probe (and containing a common component of motion in the moving condition) than other static parts of the scene which are either displaced laterally or in depth relative to the probe; (3) Rushton, Chen and Li (2018) have conducted a series of experiments using a task similar to that presented in Experiment 1 and, in a control task, showed that performance was similar after removing the possibility of making judgements relative to other parts of the environment.

### Consequences for older observers and other groups

If ability to detect scene-relative object movement during self-movement is preserved in older individuals, then it might be tempting to conclude that there is no need to further consider the mechanisms that underpin this ability or investigate how laboratory performance links to that in real-world tasks. However, as noted above (and see Figure 5), there is a good deal of variability in this performance, with some older observers significantly worse than others. It may therefore be important to identify these individuals, consider the reasons for reduced performance and investigate the consequences. Crucially, it is possible that changes in flow parsing have implications for undertaking simple activities of daily living such as driving, road crossing, or other tasks that rely on assessment of the movement of other parts of the scene while the observer is also moving. With this in mind, it will be particularly important in future work to determine whether a link can be established between metrics of flow parsing and performance in real-world tasks. Safe driving, in particular, is an activity that is likely to be highly dependent on flow parsing ability. During driving (or any other form of passive observer movement) efference copy and proprioceptive information are both downgraded in their ability to signal self-movement. Moreover, if traveling at relatively constant speed (e.g., during motorway driving) vestibular information is also likely to be less informative about self-movement. We argue therefore that it is largely as a consequence of the flow parsing mechanism that we are able to maintain ability to detect and assess object movement during driving.

So far, we have considered changes in only one aspect of the underlying global flow subtraction process that support scene-relative movement detection. However, we have also developed further tasks that examine temporal aspects of flow parsing. Rushton, Foulkes and Warren (2013) reported that the effects of global subtraction on perceived scene-relative movement were: (i) observed from as little as two frames (around 20 ms) of optic flow and (ii) were largest when the probe and flow were presented simultaneously. These findings suggest that flow parsing occurs rapidly and robustly after the onset of optic flow, as might be expected from a mechanism trying to detect scene-relative movement as quickly as possible after onset of self-movement. Given that neural processing is thought to slow in older observers (Kline & Birren, 1975; Salthouse, 1985; Salthouse, 2000; Owsley, 2013), it would be interesting and important to examine how these temporal parameters change with age and the consequences of such changes.

We might also gain further insights into flow parsing from studying other clinical and sub-clinical groups with known changes in motion processing. Schizophrenia represents one such group and Warren and Cavieres (2018) investigated flow parsing using the relative tilt task (Experiment 2) in participants with a schizophrenia (Sz) diagnosis. These data suggested a significant difference between Sz and control groups in the contributions of global versus local motion processing mechanisms to relative tilt effects. Specifically, there appeared to be higher gain on global flow subtraction relative to local motion processing mechanisms in Sz but no such difference in controls. There is some similarity, then, to the data presented here for older observers. Increased magnitude of global subtraction of optic flow might therefore be a common characteristic across groups with altered motion processing, and this might occur to preserve detection of scene relative object movement.

### Conclusions

This study indicates that ability to detect scene-relative object movement during self-movement is independent of age (at least within the broad range of ages we tested), but that there are changes in the flow-parsing mechanism that underpins this ability. We suggest that these changes might reflect the broader compensatory processing required to perceive self-movement and orientation in the face of other sensory and extraretinal impairments.

## Appendix Appendix: RANDOT vs. age and RANDOT vs. FP measures

As noted above we collected RANDOT scores simply to ensure that our participants could interpret stereoscopically presented stimuli in Experiment 1. All of our participants exhibited some level of stereoacuity, and so no participants were excluded on these grounds. Here we report the outcome of exploratory analyses to examine the relationship between RANDOT and age, which has been found in several previous studies (e.g., Zaroff, Knutelska, & Frumkes, 2003; Garnham & Sloper, 2006). In line with these studies, we found evidence for a strong positive correlation (*BF_10_* = 12.5, *r* = 0.51, *p* = 0.004) between these variables. However, there was no clear evidence for a relationship between RANDOT and the FP measures in either Experiment 1 (FPI: *BF_10_* = 0.90 *r* = 0.32, *p* = 0.090) or Experiment 2 (Opposite RelT: *BF_10_* = 0.64, *r* = 0.28, *p* = 0.142).
